# A *de novo* transcriptome assembly of the zebra bullhead shark, *Heterodontus zebra*

**DOI:** 10.1038/sdata.2018.197

**Published:** 2018-10-08

**Authors:** Koh Onimaru, Kaori Tatsumi, Kazuhiro Shibagaki, Shigehiro Kuraku

**Affiliations:** 1 Phyloinformatics unit, RIKEN Center for Life Science Technologies (CLST), 2-2-3 Minatojima-minamimachi, Chuo-ku, Kobe, Hyogo, Japan; 2 Laboratory for Phyloinformatics, RIKEN Center for Biosystems Dynamics Research (BDR), 2-2-3 Minatojima-minamimachi, Chuo-ku, Kobe, Hyogo, Japan; 3 Ibaraki Prefectural Oarai Aquarium, 8252-3, Isohama-machi, Oarai-machi, Higashiibaraki-gun, Ibaraki, Japan

**Keywords:** Transcriptomics, Molecular evolution

## Abstract

Although cartilaginous fishes have played crucial roles in various fields, including evolutionary biology, marine ecology, bioresources, and aquarium exhibitions, molecular information for these species is poorly available. The present study reports a transcriptome assembly from an embryo of the zebra bullhead shark (*Heterodontus zebra*), produced by paired-end RNA sequencing. Transcriptome data is generated with a *de novo* transcriptome assembler, Trinity. Amino acid sequences are predicted from the assemblies, using TransDecoder. Because cartilaginous fishes serve as the outgroup of bony vertebrates, the data would contribute to comparative analyses of a various biological fields. In addition, this study would be useful for conservation biology, such as transcriptome-based population genetics.

## Background & Summary

Long generation cycle, large body size, and slow growth rate are the characteristics of cartilaginous fishes^1,2^, and also the main reasons why they are difficult to keep in laboratories. These factors have distracted researchers from the modern molecular studies of cartilaginous fishes. Instead, animals with a small body and short generation time, such as fruit flies, nematodes, zebrafishes, and mice have been intensely studied as "model organisms", which has accelerated our understandings of biology^3^. However, such convenience-oriented choices of species may lead to accumulation of biased knowledge^4–6^. Indeed, recent studies showed that non-coding sequences are more comparable between the genomes of humans and cartilaginous fishes than between those of humans and zebrafishes^7–9^. This comparability is likely attributed to the slower molecular clock of cartilaginous fishes than that of teleosts^1,10,11^. Therefore, the study of cartilaginous fishes helps us recognize the secondary modifications of model vertebrate species. Because molecular information of cartilaginous fishes is currently available for a limited number of species, further augmentation of molecular data in this clade would be useful for comparative studies.

In addition, cartilaginous fishes play important roles for marine ecology, bioresources, and aquarium exhibitions^2^. Owing to the slow growth rate, long generation time, and sparse reproductive cycles, it has been realized that cartilaginous fishes are vulnerable to human impacts^2^. Therefore, an efficient and precise conservation policy is required for a sustainable interaction between humans and cartilaginous fishes. Recently, transcriptome data is increasingly utilized for population genetics, which can estimate divergence and effective population size of species^12,13^. In addition, a molecular phylogenetics-based score, “evolutionary distinctness” (ED), which evaluates species uniqueness, is also used for conservation prioritization^14,15^. In these respects, molecular information would contribute to making a more effective conservation policy for cartilaginous fishes.

**Figure 1:**
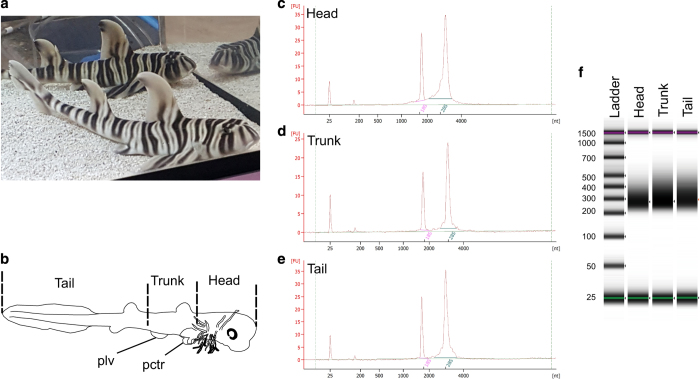
The zebra bullhead shark and sample preparation. (**a**) Juvnile zebra bullhead sharks. (**b**) A schematic diagram of a zebra bullhead shark embryo. Dashed lines, dissected positions; pctr, pectoral fins; plv, pelvic fins. (**c-e**) RNA length distribution analysis of head (**c**), trunk (**d**), and tail (**e**) samples on the 2100 Bioanalyzer, respectively. (**f**) DNA length distribution analysis of prepared libraries on the 2100 Bioanalyzer.

In this study, we report transcriptome data of the zebra bullhead shark (*Heterodontus zebra*; Fig. 1a). The zebra bullhead shark is an elasmobranch species that is common in the Western Pacific ranging from Japan to Australia^16^. The order that this species belongs to is Heterodontiformes, which includes only one living genus with nine species and relatively high ED score^17^. While the zebra bullhead shark is currently classified as Least Concern by the IUCN’s Red List, five out of the nine species are Data Deficient because their biological information is virtually missing^18^. Thus, the zebra bullhead shark may serve as a reference to characterize the species of this genus in the future. An embryo of the zebra bullhead shark was collected from Ibaraki Prefectural Oarai Aquarium. About 900,000 transcripts were assembled from the paired-end libraries of its RNAs produced by Illumina HiSeq. Of them, about 79,000 protein-coding sequences were predicted from the obtained transcript contigs.

## Methods

### Generation of the datasets

Animal experiments were conducted in accordance with the guidelines approved by the Institutional Animal Care and Use Committee (IACUC), RIKEN Kobe Branch. Zebra bullhead shark eggs were incubated at 24.5 °C, 8.0–8.2 pH in a tank of Ibaraki Prefectural Oarai Aquarium. An egg 33 days after deposition was collected, and an about 33 mm-long embryo was dissected into the head, trunk, and tail parts (Fig. 1b), and flash-frozen with liquid nitrogen, and stored at −80 °C. RNAs were extracted with the RNeasy Mini plus kit (QIAGEN, Cat. No. 74134). Genomic DNA was removed with gDNA Eliminator columns in this kit. For a quality control, the Agilent 2100 Bioanalyzer system and Agilent RNA 6000 Nano Kit (Agilent, Cat. No. 5067-1511) were used to measure their RNA integrity number, which yielded the score of 10.0 for all samples (Fig. 1c-e). For RNA-seq, using 0.5 μg of each of the extracted total RNAs, strand-specific RNA-seq libraries were prepared with the TruSeq Stranded mRNA LT Sample Prep Kit (Illumina, Cat. No. RS-122-2101 and/or RS-122-2102 ). For DNA purification, we applied 1.8x (after end repair) and 1.0x (after PCR) volumes of Agencourt AMPure XP (Beckman Coulter, Cat. No. A63880). The optimal number of PCR cycles was determined by a preliminary PCR using KAPA Library Amplification Kit (KAPA, Cat. No. KK2702) and estimated to be three cycles. The quality of the libraries was checked by Agilent 4200 TapeStation (Agilent; Fig. 1f). The libraries were sequenced after on-board cluster generation for 127 cycles using 3x HiSeq Rapid SBS Kit v2-HS (50 cycle; Illumina, Cat. No. FC-402-4022) and HiSeq PE Rapid Cluster Kit v2-HS (Illumina, Cat. No. PE-402-4002) on a HiSeq 1500 (Illumina) operated by HiSeq Control Software v2.0.12.0. The output was processed with Illumina RTA 1.18.64 for basecalling and with bcl2fastq 1.8.4 for de-multiplexing. Quality control of the obtained fastq files for individual libraries was performed with FASTQC v0.11.5. The produced data set is indicated in Table 1.

### Data processing

Using a sequence trimming pipeline, trim-galore (https://github.com/FelixKrueger/TrimGalore, version 0.4.4; parameters: --paired --phred33 -e 0.1 -q 30), adaptors and low-quality sequences were removed from the data set. To avoid contamination, we removed reads that were mapped to the genomes of other species sequenced in the same HiSeq lane (humans, mice, and the brown-banded bamboo shark), using bowtie2^19^ (version 2.2.6) to map reads and paifq (https://github.com/sestaton/Pairfq, version 0.17.0) to make pairs from unmapped reads. The overall mapping rates to other genomes were 0.11–0.12% for the human genome, 8.83–9.39% for bamboo shark genome, and 0.09–0.12% for mouse genome. This process was included because we found some contaminated transcripts in a preliminary assessment. Using a de novo transcriptome assembler, Trinity^20^ (version 2.4.0), the decontaminated reads were assembled to two initial transcriptome sets with two parameter sets: --SS_lib_type RF --trimmomatic (Assembly 1), or --SS_lib_type RF --trimmomatic --jaccard_clip (Assembly 2). Protein coding sequences (Assembly1_prot and Assembly2_prot) were predicted with a coding region finding program, TransDecoder^21^ (version 3.0.1) and using results from BlastP^22^ (2.2.31+) search against the Swissprot database^23^ and hmmscan (http://hmmer.org/, version 3.1b2) with the Pfam database (http://pfam.xfam.org/) according to the guide in TransDecoder. To reduce the complexity of the assemblies, overlapping amino acid sequences were removed from the predicted data with a clustering programme, cd-hit^24^ (parameters: -c 0.90 -n 5; Assembly1_prot_single and Assembly2_prot_single). The details of the assemblies were listed in Table 2. The commands were listed in “script.txt” in Data Citation 1.

## Data Records

The decontaminated sequence read data, which contains three records, were deposited in the NCBI Sequence Read Archive (Data Citation 2 and Table 1). The Assembly 1 was deposited at DDBJ/EMBL/GenBank (Data Citation 3 and Table 2; through the registration to the GenBank, several possible contaminants were removed from the assembly). Untrimmed reads, unfiltered Assembly 1 and 2, predicted amino acid sequences, and full quality metrics are available on figshare (Data Citation 1 and Tables 2 and 3).

## Technical Validation

Firstly, using a transcriptome quality analysis tool, TransRate^25^ (v1.0.3), we measured assembly scores and contig scores. Because this program evaluates the quality of a transcriptome assembly through mapping reads to it, we performed additional curations to the trimmed reads with trimmomatic^26^ with the same parameter set that Trinity uses (parameters: ILLUMINACLIP:$TRIMMOMATIC_DIR/adapters/TruSeq3-PE.fa:2:30:10 SLIDINGWINDOW:4:5 LEADING:5 TRAILING:5 MINLEN:25). We also modified parameters of snap-aligner^26^ and salmon^27^ in TransRate; “-h” of snap-aligner, and “--noEffectiveLengthCorrection” and “--useFSPD” of salmon were commented. The assembly scores were listed in Table 2. The program also provided “good contigs”, which were determined by the cutoff optimisation procedure described in [25]^28–31^.

Next, we evaluated the completeness of the translated assemblies, using the BUSCO programme^32^ through gVolante web server^33^. The scores were calculated with the BUSCO Vertebrata gene set^34^ and with the CVG gene set^35^ (Table 3). Overall, the completeness assessment yielded high scores for all assemblies. However, the assessment with the BUSCO Vertebrata gene set indicated slightly better completeness for Assembly 1. These figures should be interpreted carefully because the gene sets used for the assessment are mostly composed of house-keeping genes. Given the samples were obtained from a particular stage of a developing embryo, the true completeness, i.e. assembled genes/all genes that the species has, should be lower than these figures.

Because the assembly scores and the completeness scores were slightly inconsistent with each other, we also performed additional quality evaluation by examining whether the assemblies cover known genes of the horn shark (*Heterodontus francisci*), a closely related species to our target. We queried 124 genes (Data Citation 3) of the horn shark deposited in the GenBank against the translated assemblies, showing that Assembly 2 covered more known genes than Assembly 1 (Table 3). These results suggest that these two assemblies cover partially different genes. Therefore, we suggest that users need to search both of the assembles to find genes of interests.

## Additional information

**How to cite this article**: Onimaru, K. *et al*. A *de novo* transcriptome assembly of the zebra bullhead shark, *Heterodontus zebra*. *Sci. Data*. 5:180197 doi: 10.1038/sdata.2018.197 (2018).

**Publisher’s note**: Springer Nature remains neutral with regard to jurisdictional claims in published maps and institutional affiliations.

## Supplementary Material



## Figures and Tables

**Table 1:** List of raw reads.

**Organism**	**Sample**	**Protocol 1**	**Protocol 2**	**read-pairs**	**BioSample**	**Data**
Hetrodontus zebra	Embryonic head	RNA extraction	RNA-Sequencing (paired-end)	105,062,934	SAMN08388717	SRR6649877
Hetrodontus zebra	Embryonic trunk	RNA extraction	RNA-Sequencing (paired-end)	112,030,698	SAMN08388717	SRR6649876
Hetrodontus zebra	Embryonic tail	RNA extraction	RNA-Sequencing (paired-end)	103,255,692	SAMN08388717	SRR6649875

**Table 2:** Transcriptome assembly metrics.

**Assembly name**	**Source**	**Data processing**	**Coding contigs**	**BUSCOv2+vertebrates (2586 core genes)**			**BUSCOv2+CVG (233 core genes)**			**Horn shark genes (124)**	**Data Accession**
				**Complete (+partial)**	**Percentage**	**Orthologs per core genes**	**Complete (+partial)**	**Percentage**	**Orthologs per core genes**		
Assembly1_prot	Assembly1	transdecoder	189096	2496 (2553)	96.52 (98.72)	3.1	227 (233)	97.42 (100)	3.05	50^a^	figshare (Data Citation 1)
Assembly1_prot_single	Assembly1	transdecoder+cd-hit	79601	2494 (2552)	96.44 (98.69)	1.2	227 (233)	97.42 (100)	1.15	50^a^	figshare (Data Citation 1)
Assembly1_prot	Assembly2	jaccard+transdecoder	186370	2489 (2551)	96.25 (98.65)	3.05	227 (233)	97.42 (100)	3	55	figshare (Data Citation 1)
Assembly1_prot_single	Assembly2	jaccard+transdecoder+cd-hit	79383	2487 (2551)	96.17 (98.65)	1.2	227 (233)	97.42 (100)	1.15	55	figshare (Data Citation 1)

^a^Missings: AAA59375.1, AAF44636.1, AAA59377.1, AAA59373.1; too short: CAA35661.1.

**Table 3:** Completeness assessment of coding gene sets predicted from the transcriptome assemblies.

**Name**	**Libraries**	**Parameters**	**Contigs**	**Smallest**	**Largest**	**Mean length**	**n50**	**gc**	**Transrate assembly score**	**Good contig %**	**Data Accession**
Assembly 1	pooled reads from embryonic head, trunk, and tail	trimmomatic	947144	201	42242	709	1611	0.436	0.1999	88%	GenBank (Data Citation 3) figshare (Data Citation 1)
Assembly 2	pooled reads from embryonic head, trunk, and tail	trimmomatic, jaccard clip	952464	201	32662	689	1482	0.436	0.2004	68%	figshare (Data Citation 1)

## References

[d1] figshare Onimaru K. Tatsumi K. Shibagaki K. Kuraku S 2018 https://doi.org/10.6084/m9.figshare.5856912.v5 10.1038/sdata.2018.197 PMC6174923 30295671

[d2] NCBI Sequence Read Archive Onimaru K. Tatsumi K. Shibagaki K. Kuraku S 2018 SRP131715

[d3] GenBank Onimaru K. Tatsumi K. Shibagaki K. Kuraku S 2018 GGGL00000000 10.1038/sdata.2018.197 PMC6174923 30295671

